# Consumption of very low-mineral water may threaten cardiovascular health by increasing homocysteine in children

**DOI:** 10.3389/fnut.2023.1133488

**Published:** 2023-03-09

**Authors:** Yujing Huang, Yao Tan, Lingqiao Wang, Lan Lan, Jiaohua Luo, Jia Wang, Hui Zeng, Weiqun Shu

**Affiliations:** ^1^Department of Environmental Hygiene, College of Preventive Medicine, Army Medical University (Third Military Medical University), Chongqing, China; ^2^Department of Communicable Disease Prevention and Management, Chongqing Municipal Corps of Integrated Health Administrative Law Enforcement, Chongqing, China

**Keywords:** very low-mineral water, children, cardiovascular health, homocysteine, calcium, 1,25,(oh)_2_D_3_

## Abstract

**Introduction:**

Homocysteine (Hcy) is a critical factor for cardiovascular injury, and the elevation of Hcy in children will inevitably increase the risk of cardiovascular disease in adulthood. This study explored the effect of very low-mineral water on children’s Hcy and cardiovascular health.

**Materials and methods:**

This was a retrospective cohort study that recruited two groups of 10–13-year-old children who had consumed direct drinking water (DDW) in school for 4 years. The control group (NW) (119 boys, 110 girls) consumed normal DDW (conductivity 345 μs/cm). The very low-mineral water consumption group (VLW) (223 boys, 208 girls) consumed very low-mineral DDW (conductivity 40.0 μs/cm). Serum Hcy, Hcy metabolites, cofactors of Hcy metabolism, and cardiovascular biomarkers were assessed and standardized by age- and sex-specific Z-scores, and the differences between the two groups were analyzed with independent *t*-test. The relationships between Hcy metabolism biomarkers and key factors, cardiovascular biomarkers, serum Ca, and mineral intake were analyzed with linear regression.

**Results:**

Compared with the NW group, the VLW group had significantly higher serum Hcy, Apo-B, Apo-B/A1, and oxLDL, and lower serum 1,25,(OH)_2_D_3_, vitamin B6 and B12, 5-methyltetrahydrofolate, and Apo-A1. Serum Hcy was positively associated with serum Apo-B and Apo-B/A1, and negatively associated with Ca intake from water and serum 1,25,(OH)_2_D_3_.

**Conclusion:**

This study suggested that drinking very low-mineral water may increase Hcy level and oxidative stress, worsen lipid profile, and threaten the cardiovascular system in children. Reducing 1,25,(OH)_2_D_3_, and disordering of calcium metabolism might play important roles. This study first established an association between demineralized drinking water and cardiovascular health in children, suggesting a new environmental concern risk to cardiovascular health.

## 1. Introduction

Cardiovascular diseases (CVD) are a major public health problem that can lead to death and disability. Although CVD usually happens in adulthood, there is evidence that these diseases may start in childhood and adolescence (1, 2). Recently, the incidence of asymptomatic cardiovascular pathological change has increased in children. In China, the prevalence of elevated blood pressure among 6–13 years old children had risen from 14.5% (in 2010, 16.1% in boys and 12.9% in girls) to 18.4% (in 2015, 20.2% in boys and 16.3% in girls) (3). Some risk factors for CVD in childhood and adolescents are well known, such as smoking, obesity, lack of exercise, or parental history of atherosclerotic diseases (1, 2, 4). High blood homocysteine levels are found to be associated with vascular endothelial damage in children, leading to CVD in adults (5). Homocysteine (Hcy) is an amino acid occurring as an intermediate in methionine metabolism. Homocysteine increases the intracellular concentration of reactive oxygen species (ROS), disturbs the lipoprotein metabolism, increases the formation of oxidized-low density lipoprotein (oxLDL), interferes with the production of nitric oxide, induces insulin resistance, and hurts vascular endothelial cells. Given that the causal risk factor for CVD may continue from early life to adulthood, homocysteine could be considered a critical link between pre-clinical vascular changes in youth and adult CVD (6).

There are two pathways of homocysteine metabolism (7). First, homocysteine can be recycled and remethylated back to methionine, and this path needs the presence of folate and vitamin B12 (8). Second, homocysteine can be metabolized and converted into cysteine by transsulfuration, which requires vitamin B6 and cystathionine β-synthase. Numerous factors are responsible for evaluating homocysteine in the blood, including genetic abnormalities, disturbed enzyme action, and nutritional deficiencies (9). Studies on dietary factors focus on insufficient B vitamins, especially folate, vitamin B12, and vitamin B6 (8). Hyperhomocysteinemia can be caused by the deficiency of vitamin B12, B6, or folate (10, 11). Some studies revealed that drinking mineral water fortified with B vitamins could reduce blood homocysteine (12, 13). Kim et al. reported a negative association between daily calcium (Ca) intake and plasma homocysteine level (14). But no further study has explored the effect of mineral intake on homocysteine.

In our previous study, drinking very low-mineral water could increase blood homocysteine, high-sensitivity C-reactive protein (hs-CRP), and arginase levels compared with drinking tap water, and induce cardiovascular pathological lesions including interstitial edema, localized higher acidophily of cytoplasm, focal fiber dissolution, and fracture in the heart, mucoid degeneration, localized endothelial cell exfoliation, scattered foam cells, and localized intimal thickening in the intima of the aortic arch in rabbits (15). Drinking very low-mineral water could increase serum triglycerides, low-density lipoprotein, apolipoprotein (Apo)-A1, Apo B, and atherosclerosis index (Apo B/Apo A1), and decrease high-density lipoprotein in young men (15). It indicated that minerals in the water are also crucial to homocysteine metabolism and cardiovascular health.

Our previous study found that direct drinking water (DDW) on many primary school campuses are very low-mineral water resulting from the wide use of the reverse osmosis technique (16). Drinking very low-mineral water may affect children’s bone development and dental health (17, 18). Thus, children on campus also face health risks due to drinking very low-mineral water. However, there is no report about the effect of drinking very low-mineral water on children’s homocysteine metabolism and cardiovascular health. Our previous study revealed that drinking very low-mineral water may disturb the body’s Ca metabolism and decrease serum magnesium (Mg) in children, which are associated with homocysteine metabolism and cardiovascular health (17, 18). Calcium is an essential hemostatic cofactor and second messenger involved in intracellular signalings, such as the vascular system. Disturbed Ca homeostasis directly leads to increased levels of homocysteine and dyslipidemia (19). In addition, Ca may disturb mitochondrial b-oxidation and increase oxidative stress, facilitating lipid oxidation (20). Besides, consuming very low-mineral direct drinking water may decrease serum 1,25-dihydroxy vitamin D_3_ (1,25,(OH)_2_D_3_) in children in our previous study (18). 1,25,(OH)_2_D_3_ is a cofactor in the transsulfuration of homocysteine by directly upregulating cystathionine β-synthase and is negatively associated with serum Hcy (21, 22). These results support the negative role of drinking very low-mineral water in children’s homocysteine metabolism and cardiovascular system. However, studies are rare.

Considering the wild use of very low-mineral DDW on primary school campuses, investigating the effect of long-term consumption of very low-mineral water on cardiovascular health in children is necessary. This study aimed to explore the relationship between long-term consumption of very low-mineral DDW and homocysteine metabolism and its effect on cardiovascular health in children.

## 2. Methods

### 2.1. Recruiting, dietary assessment, socio-demographic characteristics collecting, and examination of the study population

This study was a continuation of our previous study (18). As we described in our previous article, it was a retrospective cohort study. The recruiting of the study population and the investigation of their dietary nutrition intake, developmental parameters, and socio-demographic characteristics were described in our previous article (18). Briefly, 1817 Han ethnic students (10–13 years old in 2013) who had annual health examinations records in 2009 were recruited from four schools that introduced the DDW systems in 2009 and had not changed them until September 2013, in Chongqing of Southwest China in March 2013.

All the subjects completed the questionnaire interview of socio-demographic characteristics (in March 2013), including sleep, socioeconomic status, outdoor exercise time outside class, medication, lifestyle factors, medical history, family history of development-associated diseases, and consumption history of mineral or vitamin supplements. Total outdoor exercise time was calculated by adding the outdoor exercise time outside class and the time for morning exercises and physical education. Participants with the following conditions were excluded: had bone fracture or transferred from other schools during 2009–2013, absented themselves from annual health examinations or summer remedial in 2013 (which lacked exposure to DDW from August to September 2013), consumed mineral or vitamin supplements, had cardiovascular disease, digestive system disease, metabolic syndrome, or development-associated diseases (hypoevolutism [assessed by height (23)], rickets, or poliomyelitis), or claimed that their family (including siblings, parents and their siblings, grandparents and their siblings) had above disease (Supplementary Figure S1). At last, 660 individuals were enrolled and completed the food records and examination in 2013. These students were divided into two groups by the mineral contents of their campus DDW. The control group (NW), including 119 boys and 110 girls, came from one school whose DDW had a mineral content close to municipal tap water (conductivity 345 μs/cm, Supplementary Table S1). The very low-mineral water consumption group (VLW) came from three schools, including 223 boys and 208 girls. The mineral content of their DDW was much lower than that of municipal tap water and lower than the recommendation by the WHO: 20 mg/l for calcium and 10 mg/L for magnesium (24) (conductivity 40.0 μs/cm, Supplementary Table S1).

The dietary mineral and nutrient, including B vitamins, intakes were obtained by 3-day food records (two weekdays and one weekend), which had been investigated twice (in March and August 2013). They were calculated according to China Food Composition (25). What is different from our previous report was that we took salt consumption into account [salt consumption data came from the study about the daily salt consumption of people in Chongqing in that period (26)].

The developmental parameters, including systolic blood pressure (SBP), diastolic blood pressure (DBP), and cardiovascular and development-associated disease information, were obtained from annual health examination records in 2009 and 2013. Ten milliliter of blood was obtained in the annual health examination in 2013 (September 2013) for the blood test.

### 2.2. Evaluation of Hcy, vitamins associated with Hcy metabolism, and biomarkers of the cardiovascular system of the study population

Serum Ca, Mg, and 1,25,(OH)_2_D_3_ were cited from our previous study (18). Serum Hcy and its metabolites [cysteine (Cys) and methionine (Met), vitamin B6, vitamin B12, 5-methyltetrahydrofolate (5-MTHF), 1,25,(OH)_2_D_3_, and biomarkers of the cardiovascular system [including Apo-A1, Apo-B, hs-CRP, vascular cellular adhesion molecule-1 (VCAM-1), soluble thrombomodulin (sTM), Resistin, OxLDL, Malondialdehyde (MDA), and 3-nitrotyrosine (3-NT)], were assessed by commercial ELISA kits (Beijing Andy Huatai technology co., LTD, Beijing, China). The ratio of Apo-B to Apo-A1 (Apo-B/A1) was calculated by Apo-B/Apo-A1.

### 2.3. Analysis of minerals in water and mineral intake from drinking water

Water samples (including tap water and DDW) were collected twice (October 2012 and May 2013). Their minerals were measured according to our previous study (16, 17, 27). Briefly, Ca and Mg were assessed by flame atomic fluorescence spectrometer. Potassium and sodium were measured by flame atomic absorption spectrophotometer (TAS-986, Purkinje General Instrument Co., Ltd., Beijing, China). Bicarbonate was assessed by indicator titration, and fluoride (F), chlorides, and sulfates were assessed by ion chromatographic method (SPD-20A, Shimadzu (China) Co., Ltd., Shanghai, China). Conductivity and pH values were detected twice per semester using a pure water tester and a pH tester (hi98308 and hi98108, Hanna Instruments, Inc., Woonsocket, RI, United States), respectively.

The mineral intake from drinking water and total mineral intake was calculated as follows:

Mineral intake from drinking water (including DDW and household drinking water, mg/d) = mineral content in DDW (mg/L) × daily consumption of DDW (L/d) + mineral content in tap water (mg/L) × daily water consumption at home (L/d).

Total mineral intake (mg/L) = mineral intake from drinking water (mg/L) + dietary mineral intake (mg/L).

### 2.4. Statistical analysis

All statistical analyses were performed with SPSS for Windows version 18.0 (SPSS Inc. Released 2009. PASW Statistics for Windows, Version 18.0. Chicago: SPSS Inc.). The independent-sample *t*-test was used to compare the difference in socio-demographic characteristics, lifestyle factors, blood pressure, and mineral intake between the two groups. Differences in the sexual distinction between the two groups were analyzed using the Chi-square test.

To avoid the influence of age and sex, standardized age- and sex-specific Z-scores of the mineral intake from drinking water and total mineral intake, serum Ca, Mg, Hcy, Cys, Met, vitamin B6, vitamin B12, 5-MTHF, 1,25,(OH)_2_D_3_, Apo-A1, Apo-1, Apo-B/A1, hs-CRP, VCAM-1, sTM, Resistin, OxLDL, MDA, and 3-NT (z-Hcy, z-Cys, z-Met, z-vitamin B6, z-vitamin B12, z-5-MTHF, z-1,25,(OH)_2_D_3_, z-Apo-A1, z-Apo-1, z-Apo-B/A1, z-hs-CRP, z-VCAM-1, z-sTM, z-Resistin, z-OxLDL, z-MDA, z-3-NT) were computed (28). The difference in Z-scores of serum Hcy, Cys, Met vitamin B6, vitamin B12, 5-MTHF, and biomarkers of the cardiovascular system was analyzed by the independent-sample *t*-test. To investigate the effects of very low-mineral DDW exposure on Hcy, Hcy metabolites, cofactors of Hcy metabolism, and biomarkers of the cardiovascular system. Hcy, Apo-A1, Apo-B, Apo-B/A1, and oxLDL were divided by their Z-scores (higher: Z-score > 0; lower: Z-score ≤ 0). Vitamin B6, B12, 5-MTHF, and Cys were divided by their Z-scores (higher: Z-score ≥ 0; lower: Z-score < 0). The effects were assessed by binary logistic regression. Effects of mineral intake and serum Ca and Mg (age- and sex-specific Z-score) on z-Hcy, z-vitamin B6, z-vitamin B12, z-5-MTHF, z-Apo-A1, z-Apo-1, z-Apo-B/A1, and z-OxLDL were assessed by single linear regression, and their combined effects were by multiple linear regression. The independent effect of serum Hcy (age- and sex-specific Z-score) on z-Apo-A1, z-Apo-1, z-Apo-B/A1, and z-OxLDL and their combined impact on mineral intake were assessed by single linear regression and multiple linear regression, respectively.

## 3. Results

### 3.1. Minerals in DDW

As we described in our previous paper (18), the mineral content and the conductivity of DDW in the school of the NW group were close to those of tap water. The mineral content of DDW in the schools of the VLW group sharply decreased compared with that of tap water and the NW group. The conductivity of the DDW is stable during 2009–2013 (Supplementary Table S1).

### 3.2. Baseline, characteristics, daily mineral, and nutrient intake of the subjects

As we described in our previous article (18), all the participants lived in the urban area. There were no significant differences in age, sex, water consumption, outdoor exercise time, sleep time, and family income between the two groups (*p* ≥ 0.05, Supplementary Table S2). There were no significant differences in height, weight, and BMI in 2009 between the two groups when the DDW (*p* ≥ 0.05, Supplementary Table S2), Children in the VLW group showed significantly lower height and weight after drinking DDW for 4 years (in 2013, *p* < 0.05, Supplementary Table S2). But the BMI in 2013 was not different between the two groups (*p* ≥ 0.05, Supplementary Table S2). There were no significant differences in SBP, DBP, and pulse pressure between the two groups when the DDW system was introduced (2009) and 4 years later (2013) (*p* ≥ 0.05, Supplementary Table S2).

There was no difference in daily dietary mineral intake, including Ca, Mg, Na, K, and Zinc, between the two groups in 2013 (*p* ≥ 0.05, Supplementary Table S3). However, a significant decrease was observed in daily Ca, Mg, and Na intake from drinking water and in total daily intake of Ca and Mg in the VLW group after the introduction of DDW (*p* < 0.01, Supplementary Table S3). There was no difference in daily intake of energy and other nutrients (protein, fat, carbohydrates, and vitamins [including thiamin, riboflavin, niacin, and folic acid]) (*p* ≥ 0.05, Supplementary Table S4).

### 3.3. Comparison of Hcy, metabolites of Hcy, and essential cofactors in Hcy metabolism between the two groups after long-term consumption of DDW

**Figure 1:**
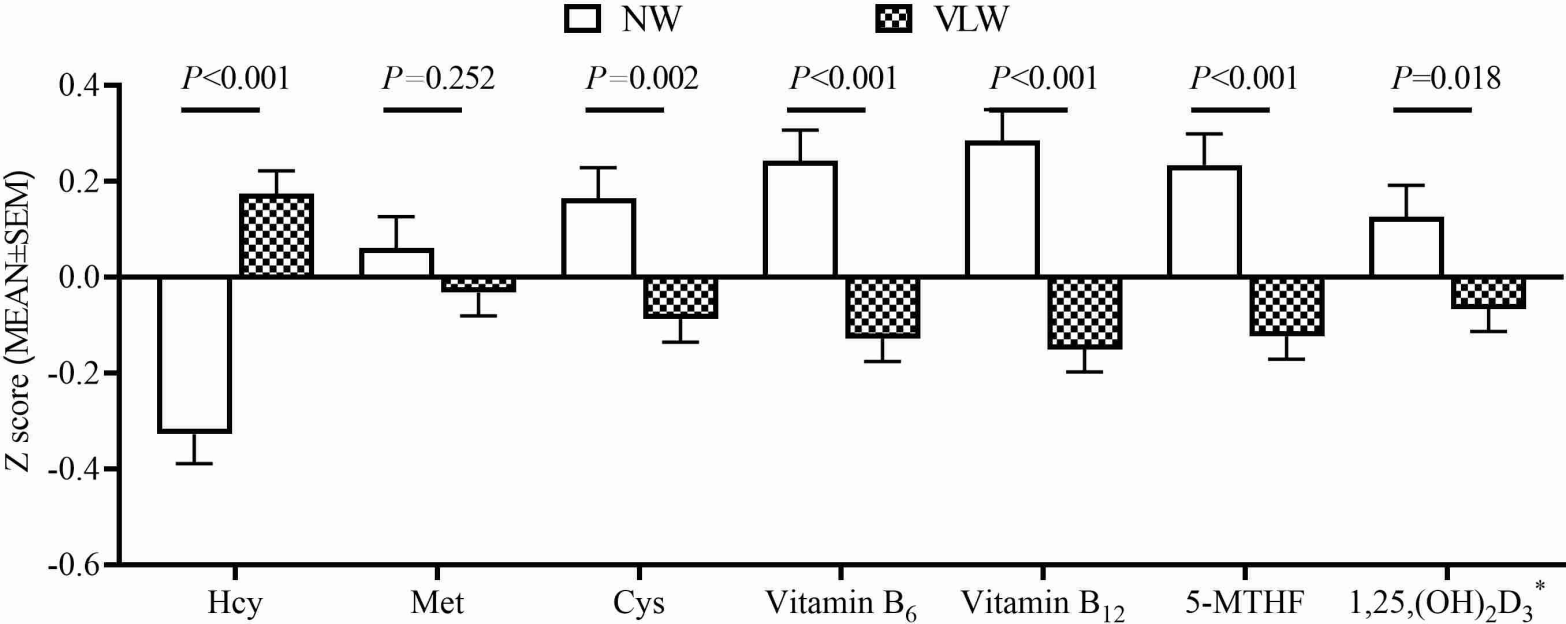
Comparison of Hcy and important associated cofactors between the two groups after consumption of DDW in children. Values were age- and sex-specific Z-scores (means ± SEM, NW: *n* = 229, VLW: *n* = 431). ^*^Data of 1,25,(OH)_2_D_3_ were cited from Huang et al. (18).

After drinking DDW for 4 years (during 2009–2013), children in the VLW group showed significantly higher z-Hcy, and lower z-Cys, z-vitamin B6, z-vitamin B12, z-5-MTHF, and z-1,25,(OH)_2_D_3_ than children in the NW group (*p* < 0.01, Figure 1). Furthermore, the higher serum Hcy, and lower serum cysteine, vitamin B6, vitamin B12, and 5-MTHF (divided by age- and sex-specific Z-scores) were positively associated with lower mineral DDW exposure (*p* < 0.01, Table 1).

**Table 1:** Associations of long-term low-mineral DDW exposure between Hcy, metabolites of Hcy, and important cofactors in Hcy metabolism.^a^

	NW	VLW	*p* value
	*n* = 229	*n* = 431	
Hcy^b^	Ref.	2.17 (1.56, 3.02)	<0.001
Cys^b^	Ref.	1.76 (1.27, 2.43)	0.001
Vitamin B6^b^	Ref.	1.71 (1.23, 2.36)	0.001
Vitamin B12^b^	Ref.	1.45 (1.05, 2.00)	0.023
5-MTHF^b^	Ref.	1.40 (1.01, 1.92)	0.042
1,25,(OH)_2_D_3_^b^^,^ ^c^	Ref.	1.36 (0.983, 1.87)	0.063

a Values were the rate ratio (95% Cl) analyzed by binary logistic regression.

b Hcy, Cys, Vitamin B6, Vitamin B12, 5-MTHF, and 1,25,(OH)_2_D_3_ were divided by their age- and sex-specific Z-scores: Hcy: 1: higher, Z-score > 0; 0: lower, Z-score ≤ 0; Cys, Vitamin B6, Vitamin B12, 5-MTHF, and 1,25,(OH)_2_D_3_: 1: lower: Z-score < 0; 0: higher: Z-score ≥ 0.

c Data of 1,25,(OH)_2_D_3_ were cited from Huang et al. (18).

### 3.4. Comparison of serum biomarkers of the cardiovascular system between the two groups after long-term consumption of DDW

**Figure 2:**
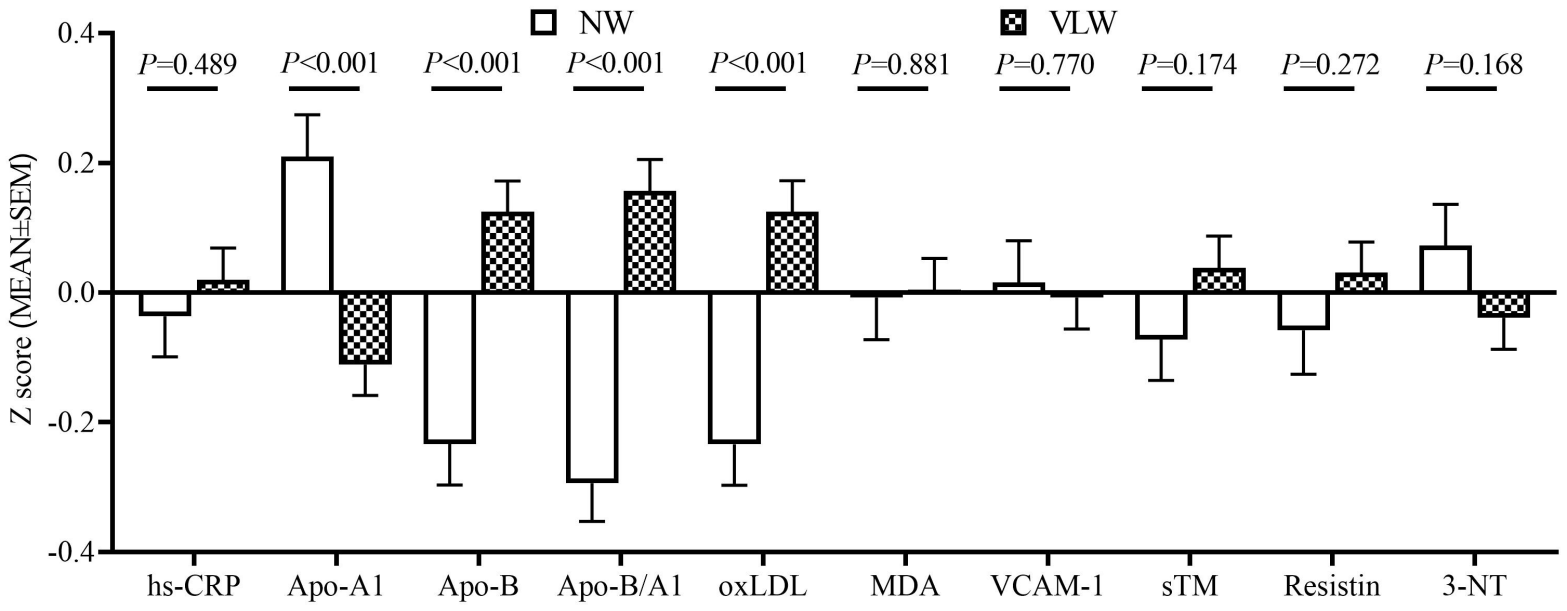
Comparison of serum biomarkers of the cardiovascular system between the two groups after consumption of DDW in children. Values were age- and sex-specific Z-scores (means ± SEM, NW: *n* = 229, VLW: *n* = 431).

After drinking DDW for 4 years (during 2009–2013), children in the VLW group showed significantly higher z-Apo-B, z-Apo-B/A1, and z-oxLDL, and lower z-Apo-A1 than children in the NW group (*p* < 0.05, Figure 2). Furthermore, the higher serum Apo-B, Apo-B/A1, and oxLDL, and lower serum Apo-A (divided by age- and sex-specific Z-scores) in the VLW group were positively associated with lower mineral DDW exposure (*p* < 0.01, Table 2).

**Table 2:** Associations between low-mineral DDW exposure and serum biomarkers of cardiovascular system.^a^

	NW	VLW	*p* value
	*n* = 229	*n* = 431	
ApoA1^b^	Ref.	1.60 (1.16, 2.21)	0.004
ApoB^b^	Ref.	1.81 (1.30, 2.50)	<0.001
Apo-B/A1^b^	Ref.	2.14 (1.53, 3.00)	<0.001
oxLDL^b^	Ref.	1.70 (1.23, 2.35)	0.001

a Values were the rate ratio (95% Cl) analyzed by binary logistic regression.

b Apo-A1, Apo-B, Apo-B/A1, and oxLDL were divided by their age- and sex-specific Z-scores: Apo-A1: 1: lower: Z-score < 0; 0: higher: Z-score ≥ 0; Apo-B, Apo-B/A1, and oxLDL: 1: higher, Z-score > 0; 0: lower, Z-score ≤ 0.

### 3.5. Associations of mineral intake with serum Hcy, metabolites of Hcy, essential cofactors in Hcy metabolism, and biomarkers of the cardiovascular system

The Ca, Mg, and Na intake from drinking water (age- and sex-specific Z-scores) was negatively associated with serum Hcy, and positively associated with serum vitamin B12 and 5-MTHF (age- and sex-specific Z-scores) (*p* < 0.05, Table 3). The Ca, Mg, and K intake from drinking water (age- and sex-specific Z-scores) was positively associated with serum vitamin B6 (age- and sex-specific Z-scores) (*p* < 0.05, Table 3). The Ca intake from drinking water (age- and sex-specific Z-scores) was positively associated with serum 1,25,(OH)_2_D_3_ (age- and sex-specific Z-scores) (*p* < 0.05, Table 3).

**Table 3:** Effects of mineral intake from daily drinking water on serum Hcy, lipid parameters, and related vitamines after consumption of DDW in children.^a^

	Ca^2^	Mg^2^	Na^2^	K^2^
Hcy^b^^,^ ^c^	−0.191 (−0.266, −0.116)^**^	−0.177 (−0.252, −0.101)^**^	−0.168 (−0.244, −0.0930)^**^	−0.0140 (−0.0900, 0.0630)
Vitamin B6^b^^,^ ^c^	0.144 (0.0680, 0.219)^**^	0.132 (0.0560, 0.208)^**^	0.00900 (−0.0680, 0.0850)	0.135 (0.0590, 0.211)^**^
Vitamin B12^b^^,^ ^c^	0.177 (0.102, 0.252)^**^	0.165 (0.0890, 0.240)^**^	0.188 (0.112,0.263)^**^	0.0180 (−0.0590, 0.0940)
5-MTHF^b^^,^ ^c^	0.162 (0.0870, 0.238)^**^	0.150 (0.0750, 0.226)^**^	0.141 (0.0650, 0.216)^**^	0.0100 (−0.0670, 0.0860)
1,25,(OH)_2_D_3_^b^^,^ ^c^^,^ ^e^	0.0780 (0.0010, 0.154)^*^	0.0710 (−0.0050, 0.147)	0.0090 (−0.0680, 0.0850)	0.0110 (−0.0660, 0.0880)
Apo-A1^b^^,^ ^c^	0.100 (0.0240, 0.176)^**^	0.0880 (0.0120, 0.165)^*^	0.117 (0.0410, 0.193)^**^	−0.0230 (−0.0990, 0.0540)
Apo-B^b^^,^ ^c^	−0.176 (−0.251, −0.100)^**^	−0.174 (−0.249, −0.0990)^**^	−0.125 (−0.201, −0.0490)^**^	−0.0990 (−0.175, −0.0230)^*^
Apo-B/A1^b^^,^ ^c^	−0.190 (−0.265, −0.115)^**^	−0.181 (−0.256, −0.106)^**^	−0.159 (−0.234, −0.0830)^**^	−0.0570 (−0.133, 0.0200)
oxLDL^b^^,^ ^c^	−0.144 (−0.219, −0.0680)^**^	−0.133 (−0.209, −0.0570)^**^	−0.141 (−0.217, −0.0650)^**^	−0.0050 (−0.0810, 0.0720)
Hcy^b^^,^ ^d^	−1.030 (−1.637, −0.424)^**^	0.846 (0.239, 1.45)^**^		
Vitamin B6^b^^,^ ^d^	0.834 (0.221, 1.45)^**^	−0.696 (−1.31, −0.0830)^*^		
Vitamin B12^b^^,^ ^d^	0.884 (0.275, 1.49)^**^	−0.712 (−1.32, −0.103)^**^		
5-MTHF^b^^,^ ^d^	0.856 (0.244, 1.47)^**^	−0.699 (−1.31, −0.0880)^*^		
1,25,(OH)_2_D_3_^b^^,^ ^d^^,^ ^e^	0.0780 (0.0010, 0.154)^*^			
Apo-A1^b^^,^ ^f^			0.117 (0.0410, 0.193)^**^	
Apo-B^b^^,^ ^f^	−0.176 (−0.251, −0.100)^**^			
Apo-B/A1^b^^,^ ^f^	−0.190 (−0.265, −0.115)^**^			
oxLDL^b^^,^ ^f^	−0.784 (−1.40, −0.171)^*^	0.646 (0.0320, 1.26)^*^		

a Values were the β (95% CI) analyzed by linear regression (*n* = 660).

b The mineral intake from daily drinking water (including DDW and household drinking water, mg/d), serum Hcy, Vitamin B6, vitamin B12, 5-MTHF, 1,25,(OH)_2_D_3_, Apo-A1, Apo-B, Apo-A1/B, and oxLDL were standardized by age- and sex-specific Z-scores.

c The association was analyzed by single linear regression (*n* = 660).

d The association was analyzed by multiple linear regression of all the factors which had a significant association in single linear regression and combined effects on Z score of serum Hcy (*n* = 660).

e Data of 1,25,(OH)_2_D_3_ were cited from: Huang et al. (18).

f The association was analyzed by multiple linear regression of all the factors which had a significant association in single linear regression (*n* = 660).

*p < 0.05; **p < 0.01.

The Ca, Mg, and Na intake from drinking water was (age- and sex-specific Z-scores) positively associated with serum Apo-A1 (age- and sex-specific Z-score), and negatively associated with Apo-B/Apo-A1 and oxLDL (age- and sex-specific Z-scores) (*p* < 0.05, Table 3). The Ca, Mg, Na, and K intake from drinking water was negatively associated with serum Apo-B (age- and sex-specific Z-score) (*p* < 0.05, Table 3). When we assessed the main factors of the four mineral intake from drinking water by multiple linear regression, the Na intake from drinking water (age- and sex-specific Z-score) was a key factor associated with z-Apo-A1 (*p* < 0.05, Table 3). Ca (age- and sex-specific Z-score) was a key factor with z-Apo-B and z-Apo-B/A1 (*p* < 0.05, Table 3). Ca and Mg intake from drinking water (age- and sex-specific Z-scores) exerted combined effects on z-Hcy and z-oxLDL (Ca: negative association; Mg: positive association; *p* < 0.05, Table 3), z-vitamin B6, z-vitamin B12, and z-5-MTHF (Ca: positive association; Mg: negative association; *p* < 0.05, Table 3). The total daily Ca intake (including drinking and diets, age- and sex-specific Z-score) was negatively associated with z-oxLDL (*p* < 0.05, Supplementary Table S5).

### 3.6. Associations of serum ca, mg, Hcy, metabolites of Hcy, essential cofactors in Hcy metabolism, and biomarkers of the cardiovascular system

The z-1,25,(OH)_2_D_3_ was negatively associated with z-Hcy [β (95% CI): −0.0885 (−0.165, −0.0123); *p* < 0.05]. The z-Hcy was positively associated with z-Apo-B and z-Apo-B/A1 (*p* < 0.05, Table 4). The z-Hcy had a combinative effect with Ca intake from drinking water (age- and sex-specific Z-scores) on z-Apo-B/A1 [β (95% CI): Ca: −0.1760 (−0.251, −0.100); Hcy: 0.0800 (0.0040, 0.157); *p* < 0.05]. Serum Ca (age- and sex-specific Z-score) was negatively associated with z-oxLDL (*p* < 0.05, Table 4). There was no association of serum Ca and Mg (age- and sex-specific Z-scores) with serum Hcy, metabolites of Hcy, and important cofactors in Hcy metabolism (age- and sex-specific Z-scores) (*p*≥0.05, Table 4 and Supplementary Table S6).

**Table 4:** Associations of serum Hcy and calcium, magnesium, 1,25,(OH)_2_D_3_ with biomarkers of cardiovascular disease in children.^a^

	Serum Hcy^b^	*p* value	Serum calcium^b^	*p* value	Serum magnesium^b^	*p* value	Serum 1,25,(OH)_2_D_3_^b^	*p* value
Apo-A1^b^	−0.0460 (−0.123, 0.0300)	0.235	−0.0220 (−0.0990, 0.0540)	0.568	0.0340 (−0.0420, 0.111)	0.382	0.0010 (−0.0760, 0.0770)	0.982
Apo-B^b^	0.100 (0.0240, 0.176)	0.010	0.0230 (−0.0530, 0.100)	0.552	0.0460 (−0.0310, 0.122)	0.240	−0.0060 (−0.0830, 0.0700)	0.877
Apo-B/A1^b^	0.114 (0.0380, 0.190)	0.003	0.0400 (−0.0370, 0.117)	0.306	0.0210 (−0.0550, 0.0980)	0.587	−0.0170 (−0.0930, 0.0600)	0.666
oxLDL^b^	0.0580 (−0.0190, 0.134)	0.140	0.0920 (0.0160, 0.168)	0.018	−0.0060 (−0.0830, 0.0700)	0.874	−0.0190 (−0.0960, 0.0570)	0.622

a Values were the β (95% CI) analyzed by single linear regression (*n* = 660).

b The serum calcium, magnesium, 1,25,(OH)_2_D_3_, Hcy, important cofactors in Hcy metabolism, and biomarkers of the cardiovascular system were standardized by age- and sex-specific Z-scores.

## 4. Discussion

In our previous study, we reported that children had seriously insufficient Ca intake from diets, which was in line with other studies (29). Drinking very low-mineral DDW aggravated children’s lack of total daily Ca and Mg intake, which disturbed Ca metabolism and decreased serum Mg level (18). But Na intake was lower than the recommendation in our previous report (775 mg/d and 780 mg/d in NW and VLW, respectively) and other studies. However, they did not estimate sodium intake reliably, resulting from the lack of salt consumption records. When we took the daily salt consumption [using the daily salt consumption of people in Chongqing in that period (26)] into account, the total daily Na intake was much higher than the recommended (more than two times). These people may have a high risk of cardiovascular disease due to their high daily Na intake (30). Drinking very low-mineral DDW cannot significantly moderate the excessive Na intake and may not benefit cardiovascular health *via* dampening the excessive Na intake.

Serum homocysteine increase has been associated with more than 100 diseases, including cardiovascular disease (5, 6). This study showed that children in the VLW group had higher serum homocysteine. In our previous study, consuming VLW increased serum Ca in children (18). Increased serum Ca can increase serum homocysteine by mediating atherothrombosis (19). But the serum homocysteine was not associated with the serum Ca in this study. The Ca intake from drinking water was a key factor for high serum homocysteine. It indicated that the homocysteine increase *via* consuming VLW might be associated with the lower Ca intake, as Tanaka reported in postmenopausal women (31). Ca plays a key role in the absorption process of vitamin B12 (32, 33). In this study, although there was no difference in the folic acid intake between the two groups, the serum vitamin B6, vitamin B12, and 5-MTHF decreased in the VLW group and were associated with Ca intake from drinking water. Furthermore, serum 1,25,(OH)_2_D_3_ was associated with serum homocysteine. 1,25,(OH)_2_D_3_ can directly upregulate cystathionine β-synthase, an enzyme involved in the transsulfuration of homocysteine, which is mediated by vitamin D receptor *via* binding together with the retinoid X receptor and acetylated histone H4 in the intergenic region of the cystathionine β-synthase gene (34). In our previous study, serum 1,25,(OH)_2_D_3_ decreased in children consuming very low-mineral water and was negatively associated with Ca intake from drinking water (18). In this study, methionine, a metabolite of homocysteine remethylating, was not different between the two groups, and cysteine, a metabolite of homocysteine transsulfuration, decreased in children drinking VLW. In another study in rats, consuming purified water decreased cystine, an oxide of cysteine, and 2-hydroxybutyric acid, the by-product of homocysteine transsulfuration (35) (data not shown). These findings supported that drinking very low-mineral water may repress homocysteine transsulfuration. Disturbing serum B vitamins and 1,25,(OH)_2_D_3_ status due to Ca deficiency in drinking water may be the key reason. Low cysteine levels will aggregate the insufficient Ca intake and Ca disorders by decreasing the absorption of vitamin D and K *via* decreasing taurine, which is synthesized from cysteine (36).

Dyslipidemia and lipid oxidation are risk factors for cardiovascular diseases (37–40). Both homocysteine metabolism and Ca homeostasis disorders can disturb lipid metabolism and induce oxidative stress (19, 20, 41). The serum Apo B, Apo B/Apo A1, and oxLDL was higher in children drinking very low-mineral DDW. Serum Apo B and Apo B/Apo A1 are biomarkers of lipid metabolism and may reflect the higher risk of atherosclerosis in adulthood (38). The serum homocysteine was associated with elevated serum Apo B and Apo B/Apo A1 in this study. And the Ca intake from drinking water had a combinative effect with serum homocysteine. Based on these findings, Ca deficiency in DDW may disturb lipid metabolism by increasing serum homocysteine, which may increase the risk of atherosclerosis in adulthood. The oxidation of LDL was considered a biomarker of oxidative stress cardiovascular system and the main atherogenic modification of LDL (42, 43). It has antigenic potential and contributes heavily to atherosclerosis-associated inflammation (44, 45). In this study, the serum oxLDL was only associated with the Ca, either the Ca intake (from the drinking water and total Ca intake) or the serum Ca level. Calcium can disturb mitochondrial b-oxidation and regulate the cellular oxidation of low-density lipoprotein by arterial smooth muscle cells (20, 46). Calcium channel blockers can significantly inhibit the increase in ox-LDL levels in Dahl salt-sensitive rats receiving a high salt diet (47). Though oxidative stress is considered the major cause of homocysteine-induced toxicity, the main pro-oxidant nature of homocysteine is forming disulfides induced by metals (40). Some reports provide that the effects of homocysteine on vascular smooth muscle cells and endothelial cells are mediated by Ca (48, 49). These indicate that deficient Ca in drinking water threatens the cardiovascular system *via* inducing lipid oxidation, which relies mainly on serum Ca disorders, rather than high homocysteine. Though the serum Ca was not associated with serum 1,25,(OH)_2_D_3_ in this study, the disturbing of bone metabolism, which increased serum Ca when Ca intake was insufficient, was associated with serum 1,25,(OH)_2_D_3_. Therefore, serum 1,25,(OH)_2_D_3_ can disturb either homocysteine metabolism or Ca metabolism, and then played a key role in cardiovascular risk by consuming very low-mineral water.

Some limitations to this study were reported in our previous paper (18). For example, we only recorded the minerals in DDW of the four schools and the dietary intake of these children in 2012 and 2013. Besides, the present study lacked data on these children’s daily vitamin B12 and B6 intake resulting from the lack of vitamin B12 and B6 contents in food in the China Food Composition (25). We cannot exclude the influence of dietary vitamins B12 and B6. But those students lived in the same area and shared similar nutritional habits. And we excluded participants who consumed mineral or vitamin supplements. We may postulate that the dietary intake of vitamin B12 and B6 was similar between the two groups. Second, these children had no symptoms or signs of cardiovascular damage, as well as higher blood pressure or hs-CRP. Children are in development, and their organs have a strong repair ability. They may not show a significant sign of cardiovascular dysfunction or morbidity. We can only use the cardiovascular biomarkers that can predict the risk of cardiovascular disease in adulthood, such as apolipoproteins and oxLDL, to evaluate the cardiovascular health of these children (38). However, we did not collect blood samples of these children in 2009. Lack of the baseline of serum homocysteine, B vitamins, and biomarkers of the cardiovascular system makes it impossible to show the causal relationship between very low-mineral water consumption and cardiovascular damage. Although the blood pressure of these children was similar in 2009, it was also similar in 2013 and cannot support the baseline of cardiovascular system status was not different (2009). Thus, more studies are required to confirm this finding.

## 5. Conclusion

This study was the first trial to evaluate the association between long-term consumption of very low-mineral water and cardiovascular health in children. We found that consumption of very low-mineral water, even though it is not the only source of daily drinking water, might disturb homocysteine and lipid metabolism, increase oxidative stress, and then threaten cardiovascular health in children. Calcium and 1,25,(OH)_2_D_3_ disorders induced by insufficient Ca intake might play a key role. Although further studies are required to confirm this finding, we still suggest that children drinking very low-mineral water or reverse-osmosis purified water are exposed to many health risks besides retarded bone development.

## Data availbility statement

The raw data supporting the conclusions of this article will be made available by the authors, without undue reservation.

## Ethics statement

The studies involving human participants were reviewed and approved by The Ethics Committee of Army Medical University, Chongqing, China. Written informed consent to participate in this study was provided by the participants’ legal guardian/next of kin.

## Author contributions

WS conceived the investigation and designed the exposure estimates. YH designed the exposure estimates, performed the experiment and statistical analyses, and drafted the manuscript. LL made substantial contributions to the data collection. YT and LW made substantial contributions to performing the experiments. JL and HZ performed the statistical analyses. JW assisted with and checked the statistical analyses and modified the manuscript. All authors contributed to the article and approved the submitted version.

## Funding

This work was supported by the National Key R&D Program of China (2021YFC3100100), the Military Major Program of People’s Liberation Army of China (Grant No. AWS18J004), and the Project of Chongqing Municipal Health Bureau (Grant No. 2012-2-447).

## Conflict of interest

The authors declare that the research was conducted in the absence of any commercial or financial relationships that could be construed as a potential conflict of interest.

## Publisher’s note

All claims expressed in this article are solely those of the authors and do not necessarily represent those of their affiliated organizations, or those of the publisher, the editors and the reviewers. Any product that may be evaluated in this article, or claim that may be made by its manufacturer, is not guaranteed or endorsed by the publisher.
