# Supplementary material for: Consumption of very low-mineral water may threaten cardiovascular health by increasing homocysteine in children
Source: Front Nutr. 2023 Mar 9;10:1133488. doi: 10.3389/fnut.2023.1133488 (PMC10034051; doi:10.3389/fnut.2023.1133488)
Supplement: Supplementary file 1 [file Data_Sheet_1.doc]

**Supplemental Material: Consumption of very low-mineral water may threaten cardiovascular health by increasing homocysteine in children**

**Author names and affiliations:**

Yujing Huang^1^, Yao Tan^1^, Lingqiao Wang^1^, Lan Lan^2^, Jiaohua Luo^1^, Jia Wang^1^, Hui Zeng^1^, Weiqun Shu^1*^

^1^ Department of Environmental Hygiene, College of Preventive Medicine, Army Medical University (Third Military Medical University), Chongqing 400038, P.R. China.

^2^Chongqing Municipal Corps of Integrated Health Administrative Law Enforcement, Chongqing 401147, P.R. China.

**Correspondence:**

Weiqun Shu, Department of Environmental Hygiene, College of Preventive Medicine, Third Military Medical University, Chongqing 400038, P.R. China. E-mail: xm0630@sina.com.

**Running Title:** Drinking low-mineral water threatens children’s cardiovascular health

# Supplementary Figures and Tables

**Supplementary Figures and Tables**

**Supplemental Table S1.** Mineral contents of school direct drinking water and tap water in 2012 and 2013.

**Supplemental Table S2.** Baseline characteristics of participants including their blood pressure before and after they consumed direct drinking water water in schools (in 2009 and 2013), and their socio-demographic characteristics and lifestyle factors in 2013.

**Supplemental Table S3.** Daily mineral intake in children consuming direct drinking water in schools for 4 y.

**Supplemental Table S4.** Daily nutrition intake in children.

**Supplemental Table S5.** Effects of daily Ca, Mg, Na, and K intake on serum Hcy, metabolites of Hcy, important cofactors in Hcy metabolism, and biomarkers of cardiovascular system after long-term consumption of DDW.

**Supplemental Table S6.** Associations of serum calcium, magnesium, 1,25,(OH)2D3, with Hcy, important cofactors in Hcy metabolism.

**Supplemental Figure S1.** Flowchart of the exclusion criteria of study participants.

**1 Supplementary Tables**

**Supplemental Table S1.** Mineral contents of school direct drinking water and tap water in 2012 and 2013^1^.

| Analyte | NW | VLW | Tap water |
| --- | --- | --- | --- |
|  | *n*=1 | *n*=3 | *n*=1 |
| Calcium, mg/L | 52.9 | 2.28±0.638 | 52.2 |
| Magnesium, mg/L | 9.94 | 0.657±0.161 | 12.5 |
| Sodium, mg/L | 8.974 | 2.190±1.40 | 12.7 |
| Potassium, mg/L | 1.00 | 0.225±0.0843 | 2.50 |
| Chlorides, mg/L | 18.3 | 3.67±0.336 | 16.0 |
| Sulfates, mg/L | 50.2 | 3.92±0.144 | 48.2 |
| Fluorides, mg/L | 0.235 | 0.0793±0.0130 | 0.200 |
| Bicarbonate, mg/L | 96.1 | 14.7±1.18 | 161 |
| Hardness, CaCO_3_, mg/L | 137 | 24.9±3.64 | 200 |
| Conductivity^2^, μS/cm | 345 | 40.0±5.60 | 409 |
| Conductivity^3^, μS/cm | 335±22.7 | 40.6±2.00 | 409 |
| pH^3^ | 7.84±0.140 | 7.49±0.0792 | 7.57 |

^1^ Values are means or means ± SEM

^2^ In 2012 and 2013.

^3^ From 2009 to 2012 (means ± SEM).

Cited from: Huang Y, Ma X, Tan Y, Wang L, Wang J, Lan L, Qiu Z, Luo J, Zeng H, Shu W: Consumption of Very Low Mineral Water Is Associated with Lower Bone Mineral Content in Children. J Nutr 2019, 149:1994-2000. doi:10.1093/jn/nxz161.

**Supplemental Table S2.** Baseline characteristics of participants, including their blood pressure before and after they consumed direct drinking water in schools (in 2009 and 2013), and their socio-demographic characteristics and lifestyle factors in 2013^1^.

|  | NW | VLW | *P* value |
| --- | --- | --- | --- |
|  | *n*=229 | *n*=431 |  |
| Age in 2009, y | 7.51±0.0757 | 7.4640.0543 | 0.647 |
| Sex |  |  | 0.956 |
| Boy | 119 (52.0) | 223 (51.7) |  |
| Girl | 110 (48.0) | 208 (48.3) |  |
| Height in 2013, cm | 151±0.441 | 146±0.336 | <0.001 |
| Weight in 2013, kg | 42.5±0.555 | 39.3±0.381 | <0.001 |
| BMI in 2013, kg/m^2^ | 18.5±0.225 | 18.3±0.157 | 0.516 |
| Height in 2009, cm | 129±0.479 | 130±0.340 | 0.521 |
| Weight in 2009, kg | 37.3±0.638 | 37.7±0.420 | 0.652 |
| BMI in 2009, kg/m^2^ | 22.3±0.354 | 22.3±0.227 | 0.861 |
| SBP in 2009, mmHg | 109±0.217 | 109 ±0.174 | 0.928 |
| DBP in 2009, mmHg | 68.3±0.446 | 67.5±0.289 | 0.124 |
| Pulse pressure in 2009, mmHg | 40.3±0.543 | 41.1±0.380 | 0.197 |
| SBP in 2013, mmHg | 103±0.583 | 104±0.412 | 0.201 |
| DBP in 2013, mmHg | 64.0±0.312 | 64.5±0.226 | 0.199 |
| Pulse pressure in 2013, mmHg | 39.4±0.310 | 39.8±0.217 | 0.272 |
| DDW consumption, L/d | 1.05±0.0188 | 1.05±0.0139 | 0.992 |
| Total water consumption, L/d | 1.56±0.0243 | 1.56±0.0181 | 0.983 |
| Ratio of DDW consumption to total water consumption, % | 0.687±0.0091 | 0.677±0.0069 | 0.402 |
| Outdoor exercise time, h/d | 1.99±0.0540 | 1.97±0.0400 | 0.698 |
| Sleep time, h/d | 9.55±0.0760 | 9.57±0.0540 | 0.788 |
| Family income, thousand yuan/person/y | 6.09 ±0.161 | 5.82±0.111 | 0.164 |

^1^ Values are means ± SEM, or number (%).

Partially cited from: Huang Y, Ma X, Tan Y, Wang L, Wang J, Lan L, Qiu Z, Luo J, Zeng H, Shu W: Consumption of Very Low Mineral Water Is Associated with Lower Bone Mineral Content in Children. J Nutr 2019, 149:1994-2000. doi:10.1093/jn/nxz161.

**Supplemental Table S3.** Daily mineral intake in children consuming direct drinking water in schools for 4 y^1^.

|  | Dietary intake | | | Intake from drinking water^2^ | | | Total intake | | | RNI (AI)^3^ |
| --- | --- | --- | --- | --- | --- | --- | --- | --- | --- | --- |
|  | NW | VLW | *P* | NW | VLW | *P* | NW | VLW | *P* |  |
|  | *n*=229 | *n*=431 |  | *n*=229 | *n*=431 |  | *n*=229 | *n*=431 |  |  |
| Ca, mg/d | 394±9.93 | 405±6.97 | 0.357 | 82.0±1.28 | 29.8±0.678 | <0.001 | 475±10.0272 | 434±7.02 | 0.001 | 1200 ^4^ |
| Mg, mg/d | 236±2.79 | 238±2.02 | 0.577 | 16.7±0.273 | 7.26±0.162 | <0.001 | 253±2.81 | 245±2.02 | 0.028 | 300 ^4^ |
| Na, mg/d | 3753±29.8 | 3749±21.2 | 0.910 | 10.7±0.168 | 4.38±0.125 | <0.001 | 3764±29.7 | 3753±21.2 | 0.774 | 1400 ^4^ |
| K, mg/d | 1617±22.2 | 1630±16.1 | 0.648 | 7.43±0.218 | 6.91±0.164 | 0.062 | 1625±22.2 | 1637±16.1 | 0.662 | 1900 ^4^ |
| Fe, mg/d | 21.5±0.345 | 21.1±0.243 | 0.377 | — | — | — | — | — | — | 15 (18) ^4^ |
| Zn, mg/d | 14.6±0.226 | 14.4±0.170 | 0.449 | — | — | — | — | — | — | 10 (9) ^4^ |
| Se, μg/d | 64.3±2.024 | 65.5±1.70 | 0.654 | — | — | — | — | — | — | 55 ^4^ |
| Cu, mg/d | 1.47±0.0567 | 1.44±0.0389 | 0.664 | — | — | — | — | — | — | 0.7 ^4^ |
| Mn, mg/d | 4.19±0.0795 | 4.25±0.0558 | 0.561 | — | — | — | — | — | — | 4 ^5^ |
| P, g/d | 1191±12.8 | 1184±8.80 | 0.634 | — | — | — | — | — | — | 0.64 ^4^ |

^1^ Values are means ± SEM.

^2^ Drinking water includes DDW and water consumed at home.

^3^ Values are recommendations for 11-y old children or 11-y old boys (girls).

^4^ RNI, recommended nutrient intake.

^5^ AI, adequate intake.

Partially cited from: Huang Y, Ma X, Tan Y, Wang L, Wang J, Lan L, Qiu Z, Luo J, Zeng H, Shu W: Consumption of Very Low Mineral Water Is Associated with Lower Bone Mineral Content in Children. J Nutr 2019, 149:1994-2000. doi:10.1093/jn/nxz161.

**Supplemental Table S4.** Daily nutrition intake in children^1^.

|  | **Dietary intake** | | | **RNI (AI)^2^** |
| --- | --- | --- | --- | --- |
|  | NW | VLW | *P* value |  |
|  | n=229 | n=431 |  |  |
| Energy, Mcal/d | 2.05±0.0184 | 2.03±0.0133 | 0.252 | 2.35 (2.05) ^3^ |
| Protein, g | 86. 9±1.10 | 84.8±0.797 | 0.128 | 60 (55) ^3^ |
| Fat, (E%) | 40.1±0.562 | 39.9±0.419 | 0.824 | 20~30^4^ |
| Carbohydrates, g/d | 225±3.73 | 225±2.64 | 0.915 | 150^4^ |
| Carbohydrates, (E%) | 44.1±0.646 | 44.6±0.474 | 0.510 | 50~60^4^ |
| Cholesterols, mg/d | 481±11.5 | 480±8.19 | 0.905 | — |
| Fiber, g/d | 6.11±0.426 | 6.81±0.383 | 0.252 | 25^4^ |
| Vitamin A, μg/d | 747±47.6 | 716±38.0 | 0.621 | 670 (630) ^3^ |
| Thiamin, mg/d | 0.988±0.0133 | 0.979±0.0096 | 0.588 | 1.3 (1.1) ^3^ |
| Riboflavin, mg/d | 1.18±0.0248 | 1.17±0.0182 | 0.668 | 1.3 (1.1) ^3^ |
| Niacin, mg/d | 23.6±0.332 | 23.3±0.257 | 0.412 | 14 (12) ^3^ |
| Vitamin C, mg/d | 64.7±1.88 | 66.2±1.33 | 0.510 | 90^3^ |
| Vitamin E, mg/d | 7.16±0.216 | 7.32±0.160 | 0.569 | 13^4^ |
| Folic acid, μg/d | 299±4.94 | 293±3.63 | 0.373 | 350^3^ |

^1^ Values are means ± SEM.

^2^ Values are recommendations for 11-y old children or 11-y old boys (girls).

^3^ RNI, recommended nutrient intake.

^4^ AI, adequate intake.

Partially cited from: Huang Y, Ma X, Tan Y, Wang L, Wang J, Lan L, Qiu Z, Luo J, Zeng H, Shu W: Consumption of Very Low Mineral Water Is Associated with Lower Bone Mineral Content in Children. J Nutr 2019, 149:1994-2000. doi:10.1093/jn/nxz161.

**Supplemental Table S5.** Effects of daily Ca, Mg, Na, and K intake on serum Hcy, metabolites of Hcy, important cofactors in Hcy metabolism, and biomarkers of the cardiovascular system after long-term consumption of DDW^1^.

|  | **Ca^2^** | **Mg^2^** | **Na^2^** | **K^2^** |
| --- | --- | --- | --- | --- |
| Hcy^2^ | -0.0080(-0.0850,0.0680) | 0.0110(-0.0660,0.0870) | -0.0340(-0.1110,0.0420) | -0.0350(-0.1110,0.0420) |
| Vitamin B6^2^ | 0.0030(-0.0740,0.0800) | -0.0400(-0.1170,0.0360) | -0.0580(-0.1340,0.0190) | -0.0060(-0.0830,0.0700) |
| Vitamin B12^2^ | 0.0310(-0.0450,0.1080) | -0.0110(-0.0870,0.0660) | 0.0130(-0.0630,0.0900) | 0.0070(-0.0700,0.0830) |
| 5-MTHF^2^ | 0.0640(-0.0130,0.1400) | 0.0150(-0.0620,0.0910) | -0.0100(-0.0870,0.0660) | 0.0210(-0.0550,0.0980) |
| 1,25,(OH)_2_D_3_^2, 3^ | 0.0410(-0.0350,0.1180) | 0.0040(-0.0720,0.0810) | -0.0170(-0.0940,0.0590) | 0.0090(-0.0680,0.0850) |
| Apo-A1^2^ | -0.0120(-0.0890,0.0650) | -0.0030(-0.0790,0.0740) | -0.0020(-0.0790,0.0740) | -0.0060(-0.0830,0.0700) |
| Apo-B^2^ | 0.0000(-0.0760,0.0770) | 0.0660(-0.0100,0.1430) | 0.0580(-0.0180,0.1350) | 0.0130(-0.0630,0.0900) |
| Apo-B/A1^2^ | 0.0340(-0.0420,0.1110) | 0.0720(-0.0040,0.1480) | 0.0670(-0.0100,0.1430) | 0.0280(-0.0480,0.1050) |
| oxLDL^2^ | -0.0950(-0.1720,-0.0190)* | -0.0470(-0.1240,0.0290) | -0.0470(-0.1240,0.0290) | -0.0760(-0.1530,0.0000) |

^1^ Values are the β (95% CI) analyzed by single linear regression (n= 660).

^2^ The total daily mineral intake (including drinking and diets), serum Hcy, vitamin B6, vitamin B12, 5-MTHF, 1,25,(OH)_2_D_3_, Apo-A1, Apo-B, Apo-A1/B, and oxLDL were standardized by age- and sex-specific Z-scores.

^3^Data of 1,25,(OH)_2_D_3_ were cited from: Huang Y, Ma X, Tan Y, Wang L, Wang J, Lan L, Qiu Z, Luo J, Zeng H, Shu W: Consumption of Very Low Mineral Water Is Associated with Lower Bone Mineral Content in Children. J Nutr 2019, 149:1994-2000. doi:10.1093/jn/nxz161.

**P*<0.05

**Supplemental Table S6.** Associations of serum calcium, magnesium, 1,25,(OH)_2_D_3_, with Hcy, important cofactors in Hcy metabolism^1^.

|  | **Serum calcium^2^** | ***P* value** | **Serum magnesium^2^** | ***P* value** | **Serum 1,25,(OH)_2_D_3_^2,3^** | ***P* value** |
| --- | --- | --- | --- | --- | --- | --- |
| Hcy^2^ | -0.0050(-0.0820,0.0710) | 0.892 | 0.0400(-0.0360,0.1170) | 0.304 | -0.0880(-0.1650,-0.0120) | 0.023 |
| Vitamin B6^2^ | -0.0490(-0.1250,0.0280) | 0.214 | 0.0090(-0.0680,0.0850) | 0.82 | 0.0520(-0.0250,0.1280) | 0.184 |
| Vitamin B12^2^ | 0.0060(-0.0710,0.0820) | 0.883 | 0.0480(-0.0290,0.1240) | 0.221 | -0.0190(-0.0950,0.0580) | 0.634 |
| 5-MTHF^2^ | -0.0010(-0.0770,0.0760) | 0.983 | -0.0340(-0.111,0.0420) | 0.379 | 0.0260(-0.0500,0.1030) | 0.499 |
| 1,25,(OH)_2_D_3_^3^ | -0.0100(-0.0860,0.0670) | 0.801 | -0.0400(-0.117,0.0360) | 0.301 | — | — |

^1^ Values are the β (95% CI) analyzed by single linear regression (n= 660).

^2^ The serum calcium, magnesium, 1,25,(OH)_2_D_3_, Hcy, vitamin B6, vitamin B12, and 5-MTHF were standardized by age- and sex-specific Z-scores.

^3^Data of 1,25,(OH)_2_D_3_ were cited from: Huang Y, Ma X, Tan Y, Wang L, Wang J, Lan L, Qiu Z, Luo J, Zeng H, Shu W: Consumption of Very Low Mineral Water Is Associated with Lower Bone Mineral Content in Children. J Nutr 2019, 149:1994-2000. doi:10.1093/jn/nxz161.

**2 Supplementary Figures**


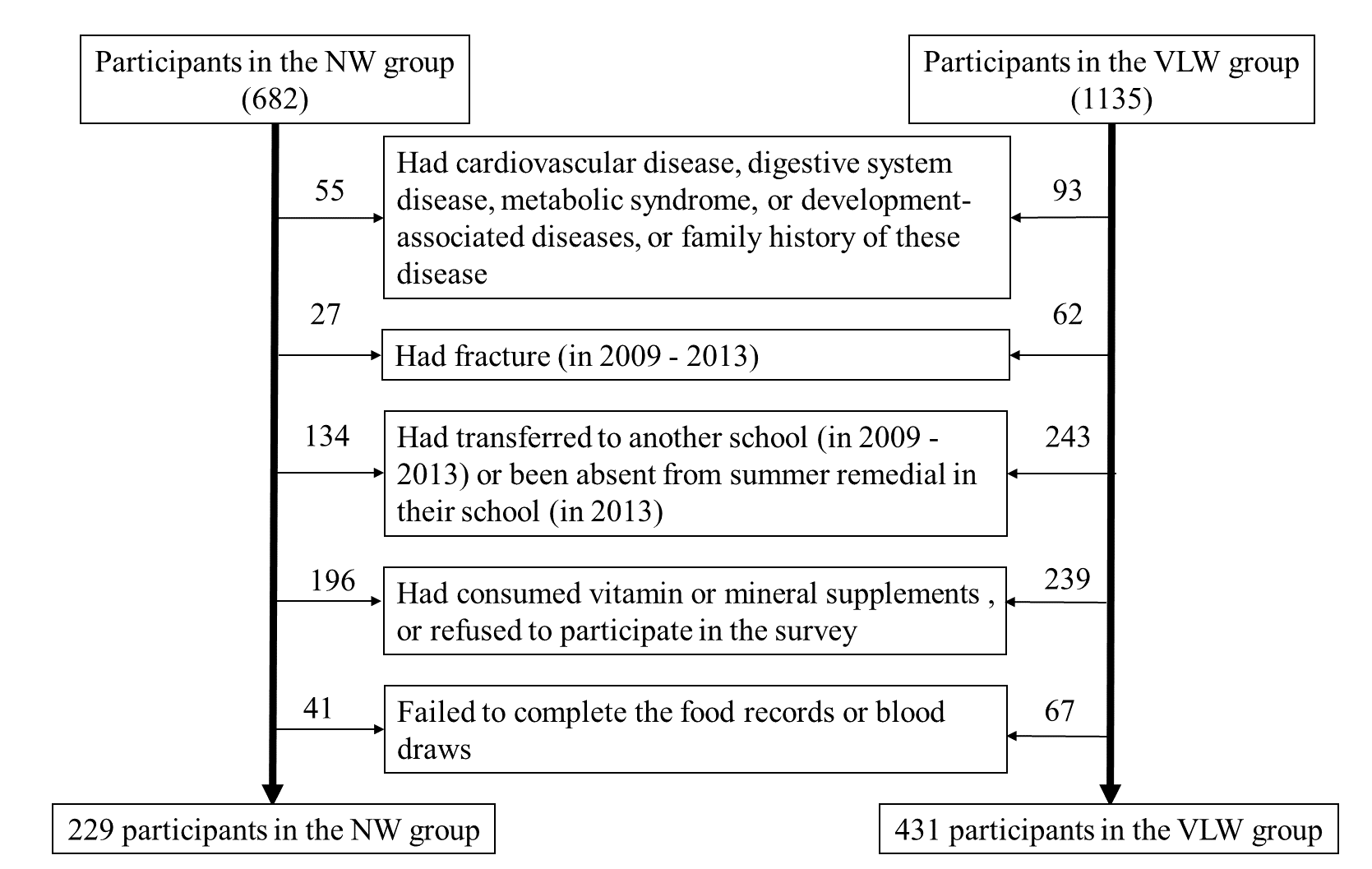


**Supplemental Figure S1. Flowchart of the exclusion criteria of study participants.**

Cited from: Huang Y, Ma X, Tan Y, Wang L, Wang J, Lan L, Qiu Z, Luo J, Zeng H, Shu W: Consumption of Very Low Mineral Water Is Associated with Lower Bone Mineral Content in Children. J Nutr 2019, 149:1994-2000. doi:10.1093/jn/nxz161.
